# A randomized controlled clinical trial examining the effects of *Cordyceps militaris* beverage on the immune response in healthy adults

**DOI:** 10.1038/s41598-024-58742-z

**Published:** 2024-04-05

**Authors:** Atcharaporn Ontawong, Sirinat Pengnet, Arthid Thim-Uam, Narongsuk Munkong, Nukrob Narkprasom, Kanjana Narkprasom, Kullanat Kuntakhut, Natakorn Kamkeaw, Doungporn Amornlerdpison

**Affiliations:** 1 Division of Physiology, School of Medical Sciences, University of Phayao, Phayao, 56000 Thailand; 2 Division of Biochemistry, School of Medical Sciences, University of Phayao, Phayao, 56000 Thailand; 3 Department of Pathology, School of Medicine, University of Phayao, Phayao, 56000 Thailand; 4 Division of Food Engineering, Faculty of Engineering and Agro-Industry, Maejo University, Chiang Mai, 50290 Thailand; 5 Center of Excellence in Agricultural Innovation for Graduate Entrepreneur, Maejo University, Chiang Mai, 50290 Thailand; 6 Faculty of Fisheries Technology and Aquatic Resources, Maejo University, Chiang Mai, 50290 Thailand

**Keywords:** *Cordyceps militaris*, Cordycepin, Functional beverage, Immunomodulation, NK activity, Disease prevention, Outcomes research

## Abstract

*Cordyceps militaris* (L.) Link (*C. militaris*) contains various beneficial substances, including polysaccharides (galactomannan), nucleotides (adenosine and cordycepin), cordycepic acid, amino acids, and sterols (ergosterol and beta-sitosterol). It also contains other essential nutrients, such as protein, vitamins (E, K, B1, B2, and B12), and minerals (potassium, sodium, calcium, magnesium, iron, zinc, and selenium). Due to the numerous health benefits of supplements and products containing *C. militaris* extract, their popularity has increased. However, the immunostimulant effect of *C. militaris* remains unclear. Therefore, this study developed a functional beverage from the submerged fermentation of *C. militaris* (FCM) and aimed to investigate the potential of FCM in healthy male and female volunteers in Phayao Province, Thailand. This study provides essential information for the development of healthy drink products. Healthy men and women were provided either FCM containing 2.85 mg of cordycepin or placebo for 8 weeks (n = 10 for each gender). The immune cell markers, immunoglobulins, and safety parameters were assessed initially at baseline and at 4 and 8 weeks. The NK cell activity markedly increased in the male FCM group from baseline (*p* = 0.049) to 4 weeks after receiving FCM. Compared with those in the placebo group, the NK activity in women who received FCM for 8 weeks significantly increased (*p* = 0.023) from baseline. Within-group analysis revealed that the IL-1β levels were markedly reduced in the male FCM group (*p* = 0.049). Furthermore, the IL-6 levels decreased from baseline in the female FCM group (*p* = 0.047). The blood sugar, lipid, and safety indices were not different between the experimental groups. FCM can potentially be developed as an immune-boosting supplement without liver, kidney, or blood component toxicity.

## Introduction

The primary function of the immune system is to protect against pathogens or foreign substances^1^. The immune response eliminates pathogens, xenobiotics, and any toxic molecules they synthesize^2^. T lymphocyte cells produce cytokines and chemokines such as IL-1, TNF-α, and IL-6 during an immune response. These cytokines induce B lymphocyte cell differentiation. B cells attach to foreign antigens to induce immune stimulation and secrete immunoglobulins^3^. Several studies have reported that cytokines affect various inflammatory diseases, including autoimmune diseases, cardiovascular diseases, gastrointestinal disorders, and lung diseases. In addition, inflammatory-related diseases are the cause of more than 50% of deaths worldwide^4^. Thus, supplements that can reduce risk factors for inflammatory-related disease and support the immune system are urgently needed.

*Cordyceps militaris* (L.) Link (*Cordyceps militaris*) is a traditional Chinese medicine (TCM) and has been officially recognized as an herbal drug by the Chinese Pharmacopoeia since 1964. Cordyceps are rich in various beneficial substances, including polysaccharides (galactomannan), nucleotides (cordycepin and adenosine), cordycepic acid, amino acids, and sterols (ergosterol and beta-sitosterol)^5^. *Cordyceps militaris* has shown strong anti-inflammatory, antioxidant, neuroprotective, antiaging, antitumor, antiproliferation, antimetastatic, and immunomodulatory effects^6,7^. Water extract of *C. militaris* at 20 mg/kg body weight increases interferon (IFN)-γ secretion^8^. Moreover, *C. militaris* aqueous extract suppressed asthma in an ovalbumin (OVA)-induced asthma mouse model^9^. At 500 mg/kg body weight, *C. militaris* reduced diarrhea and gastrointestinal bleeding in mice with dextran sodium sulfate-induced acute colitis^10^. Treatment of mice with polysaccharides from *C. militaris* markedly increased thymic and splenic indices in a dose-dependent manner^11,12^. Similarly, polysaccharides extracted from *C. militaris* reduced lung inflammatory cytokine and IgE levels in allergic asthma mice^13^. *C. militaris* is also involved in an allergic mechanism; fermented *C. militaris* markedly activated anti-allergic responses by inhibiting immunoglobulin E (IgE)/antigen-induced degranulation in RBL-2H3 cells^14^.

**Figure 1:**
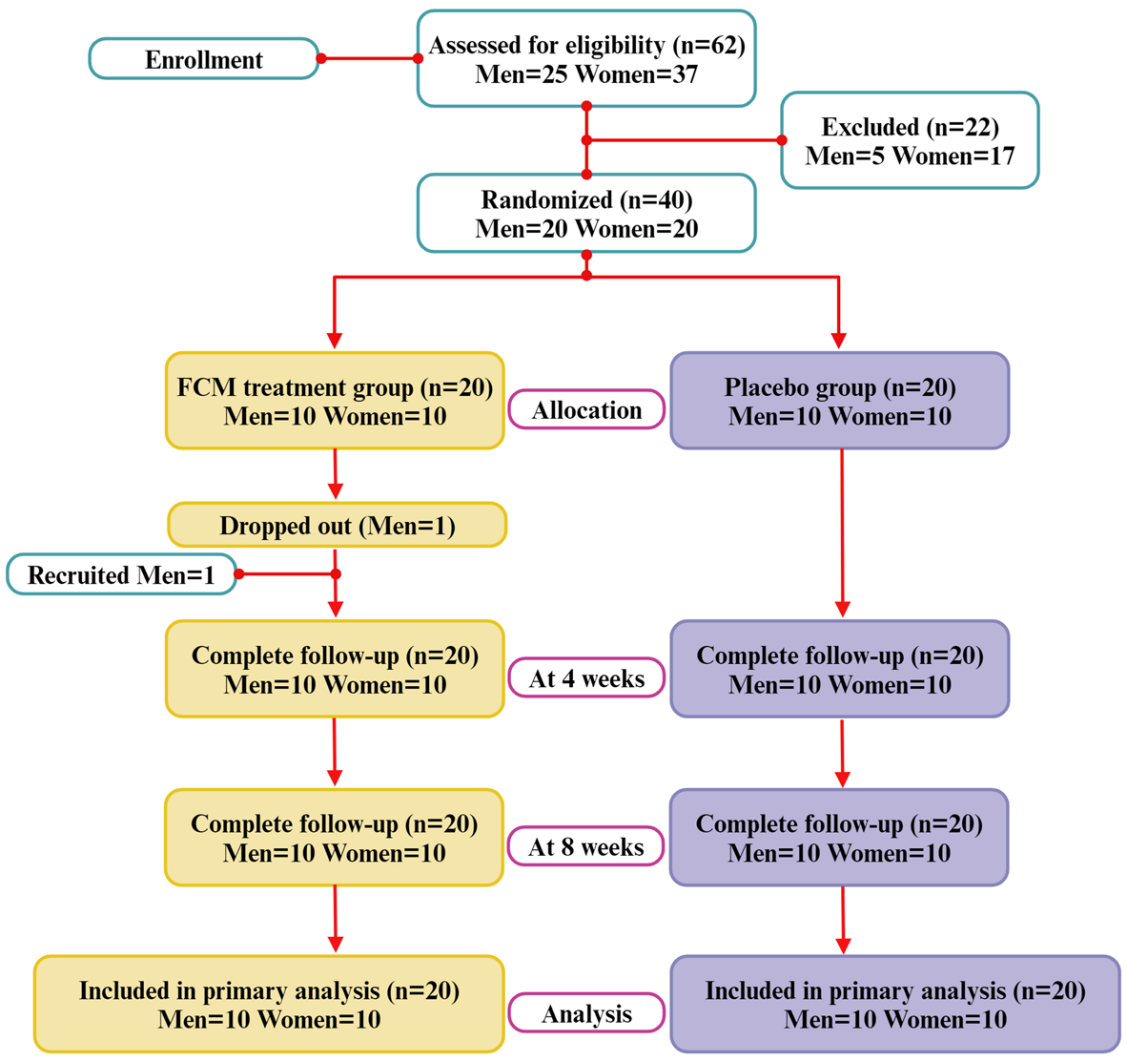
Diagrammatic synopsis of the participant enrollment process.

Furthermore, healthy volunteers received *C. militaris* capsules containing 32% cordyceps polysaccharide, 7.3% cordycepic acid, 0.13% adenosine, and 0.001% cordycepin to stimulate NK cell activity, suggesting that *C. militaris* could be used as an immune activator^7^. In addition, Korean men who received 1.5 g/day *C. militaris* showed a statistically significant increase in NK200, lymphocyte PI, IL-2, and IFN-c levels compared with the basal level in the *C. militaris* group compared with the placebo group^15^. Due to the numerous health benefits of supplements and products containing *C. militaris* extract, their popularity has increased. Moreover, we developed a functional beverage from submerged fermentation of *C. militaris* (FCM). However, clinical studies regarding the immunomodulatory effects of FCM in patients of each gender are lacking. Therefore, this study aimed to investigate the immunostimulatory effects of FCM in healthy male and female volunteers in Phayao Province to provide basic information for the development of affordable health drink products (Fig. 1).

## Results

### Standardization of *C. militaris* mycelium

**Figure 2:**
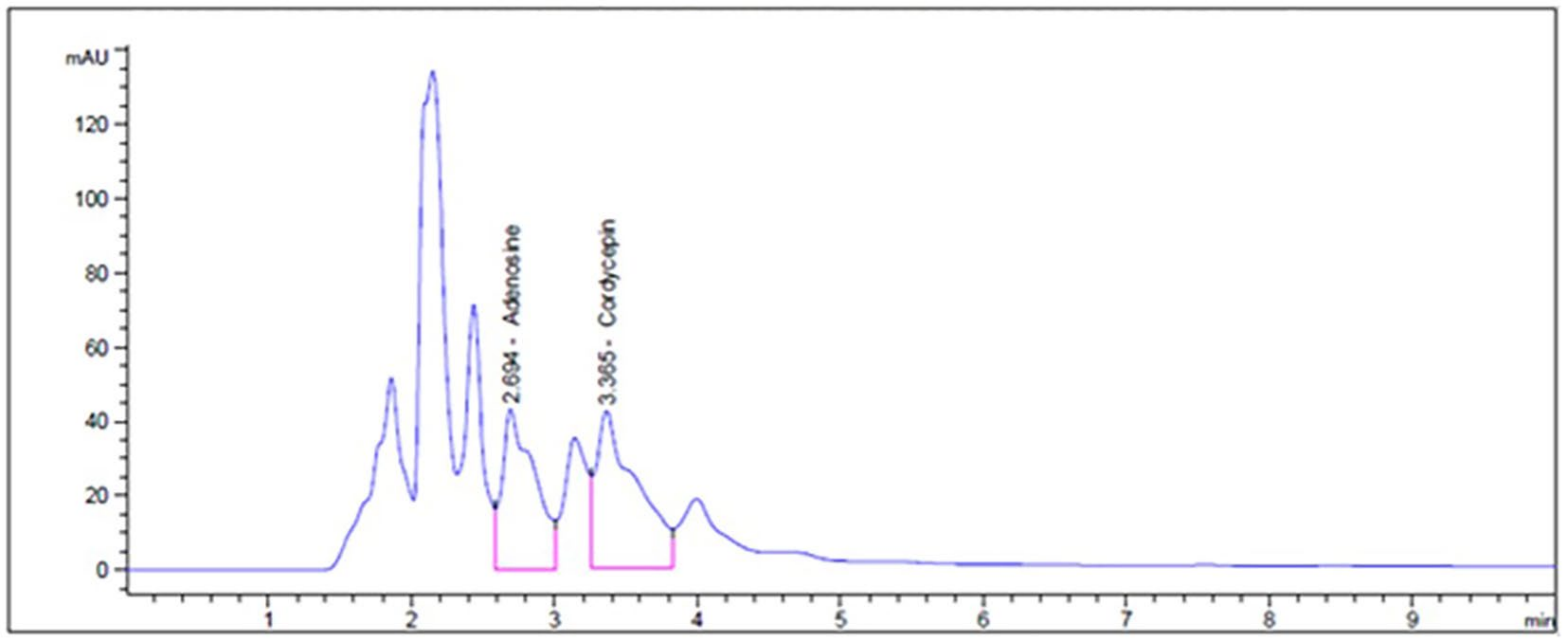
A representative HPLC chromatogram of *C. militaris* mycelium powder.

The active compounds in *C. militaris* mycelium were detected using HPLC–DAD. The chromatogram in Fig. 2 presents retention times of 2.694 min for cordycepin and 3.365 min for adenosine. The *C. militaris* powder contains 1373.21 mg/100 g of cordycepin and 511.15 mg/100 g of adenosine.

### General characteristics of the participants

Sixty-two participants were evaluated for eligibility, and 40 participants (20 men and 20 women) met the inclusion criteria (Fig. 1). Twenty-two participants, including those with hyperlipidemia, hypertension, or high levels of plasma alanine transaminase (ALT) and aspartate transaminase (AST), were excluded from this study because they did not meet the inclusion criteria. One week after the experiment began, one male participant was excluded because he was unable to participate. Thus, we recruited one more male participant. The ages and general characteristics of the 40 participants were not significantly different between the treated and placebo groups for either men or women, as shown in Table 1.

**Table 1:** General characteristics of the participants.

	Men			Women		
	FCM supplemented group (n = 10) mean ± SD	Placebo group (n = 10) mean ± SD	p value*	FCM supplemented group (n = 10) mean ± SD	Placebo group (n = 10) mean ± SD	p value*
Age (years)	41.50 ± 10.37	37.29 ± 10.58	0.259	39.40 ± 7.71	40.40 ± 5.36	0.620
Height (cm)	167.10 ± 11.17	169.00 ± 7.68	0.806	155.50 ± 4.95	157.20 ± 4.71	0.269
Weight (kg)	64.75 ± 9.62	68.21 ± 8.15	0.380	54.96 ± 7.39	55.15 ± 6.33	1.000
Body mass index; BMI (kg/m^2^)	23.16 ± 2.23	23.88 ± 2.49	0.495	22.87 ± 4.01	22.34 ± 2.53	0.821
Vital signs						
SBP (mmHg)	124.00 ± 10.97	127.00 ± 7.96	0.591	121.80 ± 11.64	117.60 ± 10.47	0.520
DBP (mmHg)	82.70 ± 10.01	87.13 ± 8.32	0.434	82.90 ± 7.91	82.10 ± 9.41	0.677
Pulse rate (bpm)	83.50 ± 14.11	79.71 ± 8.81	0.696	83.00 ± 10.47	76.70 ± 6.78	0.120
Respiratory rate (bpm)	18.80 ± 3.29	21.71 ± 3.90	0.123	20.80 ± 3.16	76.70 ± 3.16	0.802

Values are presented as the mean ± SD.

### Hematological indices

The effects of FCM on the complete blood count (CBC) parameters, including the red blood cell (RBC) count, hemoglobin (Hb), hematocrit (HCT), mean corpuscular volume (MCV), mean corpuscular hemoglobin (MCH), mean corpuscular hemoglobin concentration (MCHC), red cell distribution width (RDW), and white blood cell (WBC), neutrophil, lymphocyte, monocyte, eosinophil, basophil, and platelet counts, in male participants are shown in Table 2. The results showed that the percentage of monocytes was increased in the FCM group at weeks 4 and 8 (*p* = 0.028 and 0.009, respectively) after supplementation compared with baseline. However, there was no significant difference in CBC parameters between groups of female participants, as shown in Table 3.

**Table 2:** Comparison of the hematological indices in serum samples from men supplemented with FCM and the placebo group at weeks 0, 4, and 8.

Outcome variable	FCM supplemented group (n = 10) mean ± SD (CI)		Placebo group (n = 10) mean ± SD (CI)		*P* value^#^
RBC count (× 10^6^/μL)		p value*		p value*	
Baseline	5.20 ± 0.41 (4.95–5.44)		5.49 ± 0.47 (5.21–5.76)		0.140
4 wk	5.25 ± 0.43 (5.00–5.51)	0.623	5.61 ± 0.49 (5.32–5.90)	0.623	0.178
8 wk	5.12 ± 0.52 (4.82–5.43)	0.970	5.48 ± 0.47 (5.20–5.75)	1.000	0.199
Hb (g/dL)					
Baseline	13.76 ± 1.21 (13.05–14.47)		14.08 ± 1.06 (13.45–14.71)		0.212
4 wk	14.13 ± 1.41 (13.30–14.96)	0.472	14.54 ± 0.86 (14.40–15.04)	0.198	0.566
8 wk	13.71 ± 1.34 (12.92–14.50)	0.820	14.08 ± 1.05 (13.47–14.69)	0.879	0.384
HCT (%)					
Baseline	42.20 ± 3.08 (40.39–44.01)		43.40 ± 2.63 (41.85–44.95)		0.138
4 wk	42.80 ± 3.61 (40.67–44.95)	0.760	44.50 ± 2.88 (42.81–46.19)	0.400	0.189
8 wk	41.80 ± 3.88 (39.52–44.08)	0.703	43.40 ± 3.24 (39.52–44.08)	0.848	0.238
MCV (fL)					
Baseline	81.70 ± 8.30 (76.82–86.58)		79.70 ± 8.27 (74.84–84.56)		0.677
4 wk	81.90 ± 8.25 (77.05–86.75)	0.909	79.90 ± 7.80 (24.55–27.65)	0.909	0.652
8 wk	82.30 ± 8.47 (77.32–87.28)	0.820	79.60 ± 7.75 (75.04–84.16)	0.909	0.519
MCH (pg)					
Baseline	26.60 ± 3.20 (24.72–28.48)		25.80 ± 3.16 (23.94–27.66)		0.619
4 wk	27.00 ± 2.94 (25.27–28.73)	0.849	26.10 ± 2.64 (24.55–27.65)	0.939	0.457
8 wk	26.90 ± 3.03 (25.12–28.68)	0.909	25.90 ± 2.81 (24.25–27.55)	1.000	0.518
MCHC (g/dL)					
Baseline	32.70 ± 0.67 (32.30–33.10)		32.40 ± 0.97 (31.83–32.97)		0.490
4 wk	33.00 ± 0.82 (32.52–33.48)	0.849	32.60 ± 0.52 (32.30–32.90)	0.939	0.344
8 wk	32.90 ± 0.57 (32.57–33.23)	0.909	32.50 ± 0.71 (32.08–32.92)	1.000	0.131
RDW (%)					
Baseline	13.96 ± 1.03 (13.36–14.56)		14.27 ± 0.93 (13.72–14.82)		0.426
4 wk	14.22 ± 1.15 (13.54–14.90)	0.518	14.79 ± 0.85 (14.29–15.29)	0.204	0.513
8 wk	14.06 ± 0.49 (13.77–14.35)	0.820	14.63 ± 0.76 (14.18–15.08)	0.255	0.068
WBC count (× 10^3^/μL)					
Baseline	6.76 ± 0.87 (6.25–7.27)		8.44 ± 2.62 (6.90–9.98)		0.140
4 wk	7.50 ± 2.07 (6.28 ± 8.72)	0.343	7.58 ± 1.04 (6.97–8.19)	0.427	0.220
8 wk	6.77 ± 1.39 (5.95–7.59)	0.910	7.76 ± 2.04 (6.56–8.96)	0.405	0.273
Neutrophil (%)					
Baseline	49.40 ± 5.64 (46.08–52.72)		53.50 ± 11.51 (46.73–60.27)		0.121
4 wk	49.60 ± 8.71 (44.48–54.72)	0.910	48.00 ± 9.19 (42.60–53.40)	0.120	0.513
8 wk	45.40 ± 9.81 (39.63–51.17)	0.140	48.20 ± 9.87 (42.39–54.01)	0.211	0.427
Lymphocyte (%)					
Baseline	41.10 ± 5.38 (37.93–44.27)		37.60 ± 9.78 (31.85–43.35)		0.139
4 wk	39.00 ± 9.17 (33.61–44.39)	0.427	39.80 ± 8.16 (35.00–44.60)	0.364	0.713
8 wk	39.90 ± 9.05 (34.58–45.22)	0.940	41.70 ± 10.20 (35.70–47.70)	0.343	0.762
Monocyte (%)					
Baseline	3.33 ± 1.80 (2.28–4.39)		4.00 ± 1.56 (3.08–4.92)		0.293
4 wk	5.50 ± 2.59 (3.98–7.02)	0.028*	5.30 ± 2.45 (3.86–6.74)	0.216	0.967
8 wk	6.80 ± 3.52 (4.73–8.87)	0.009*	5.00 ± 3.13 (3.16–6.48)	0.466	0.223
Eosinophil (%)					
Baseline	3.60 ± 1.78 (2.56–4.64)		3.33 ± 1.32 (2.56–4.11)		0.398
4 wk	3.80 ± .94 (2.07–5.53)	0.969	3.50 ± 1.51 (1.62–4.63)	0.511	0.869
8 wk	3.60 ± 2.17 (2.32–4.88)	0.969	3.44 ± 1.33 (2.67–4.22)	0.728	0.463
Basophil (%)					
Baseline	1.25 ± 0.46 (0.98–1.52)		1.50 ± 1.00 (0.96–2.04)		0.118
4 wk	1.75 ± 0.96 (1.24–2.26)	0.224	1.60 ± 0.89 (1.10–2.10)	0.614	0.855
8 wk	1.29 ± 0.49 (1.01–1.57)	0.737	1.43 ± 0.53 (1.12–1.74)	0.193	0.776
Platelet count; PLT (× 10^3^/μL)					
Baseline	234.70 ± 35.47 (213.84–255.56)		225.80 ± 48.22 (197.44–254.16)		0.496
4 wk	227.20 ± 35.13 (206.54–247.86)	0.705	211.50 ± 40.98 (245.94–286.46)	0.473	0.462
8 wk	224.50 ± 31.25 (206.13–242.87)	0.520	208.20 ± 44.42 (182.08–234.32)	0.364	0.241

Values are presented as the mean ± SD (CI).

*P < 0.05, compared with the baseline within the group.

**Table 3:** Comparison of the levels of hematological indices in serum samples of women supplemented with FCM and the placebo group at weeks 0, 4, and 8.

Outcome variable	FCM supplemented group (n = 10) mean ± SD (CI)		Placebo group (n = 10) mean ± SD (CI)		*P* value^#^
RBC count (× 10^6^/μL)		p value*		p value*	
Baseline	4.83 ± 0.45 (4.57–5.10)		4.80 ± 0.48 (4.52–5.08)		0.762
4 wk	4.77 ± 0.27 (4.61–4.93)	0.677	4.83 ± 0.35 (4.62–5.03)	0.705	0.341
8 wk	4.85 ± 0.33 (4.82–5.43)	0.940	4.81 ± 0.47 (4.53–5.08)	0.970	0.850
Hb (g/dL)					
Baseline	13.04 ± 1.06 (12.42–13.66)		13.05 ± 0.64 (12.67–13.43)		0.880
4 wk	12.88 ± 0.85 (12.38–13.38)	0.677	13.24 ± 0.64 (12.86–13.62)	0.361	0.216
8 wk	13.00 ± 0.68 (12.60–13.40)	0.970	13.17 ± 0.68 (12.77–13.57)	0.705	0.519
HCT (%)					
Baseline	39.80 ± 4.05 (37.42–42.18)		40.10 ± 2.42 (38.67–41.53)		0.675
4 wk	39.30 ± 2.50 (37.83–40.77)	0.879	40.50 ± 1.72 (39.49–41.51)	0.566	0.177
8 wk	40.10 ± 1.91 (38.98–41.22)	0.676	40.30 ± 2.31 (38.94–41.66	0.939	0.878
MCV (fL)					
Baseline	82.30 ± 4.74 (79.51–85.09)		84.10 ± 5.28 (81.00–87.20)		0.471
4 wk	82.60 ± 4.60 (79.90–85.30)	0.849	84.00 ± 5.14 (80.98–87.02)	0.909	0.480
8 wk	82.70 ± 4.85 (79.85–85.55)	0.761	84.40–5.44 (81.20–87.60)	0.849	0.569
MCH (pg)					
Baseline	27.00 ± 2.00 (25.82–28.18)		27.30 ± 2.21 (26.00–28.60)		0.789
4 wk	27.00 ± 1.94 (25.86–28.14)	1.000	27.40 ± 2.01 (26.22–28.58)	0.878	0.693
8 wk	27.10 ± 2.02 (25.91–28.29)	0.908	27.50 ± 2.27 (26.16–28.84)	0.789	0.731
MCHC (g/dL)					
Baseline	33.10 ± 0.99 (32.52–33.68)		32.50 ± 0.71 (32.08–32.92)		0.203
4 wk	27.00 ± 1.94 (25–86-28.14)	1.000	27.40 ± 2.01 (26.22–28.58)	0.878	0.893
8 wk	32.30 ± 0.82 (31.82–32.78)	0.908	32.70 ± 0.67 (32.30–33.10)	0.789	0.341
RDW (%)					
Baseline	13.41 ± 0.94 (12.86–13.96)		13.81 ± 0.80 (13.34–14.28)		0.150
4 wk	13.77 ± 0.53 (13.46–14.08)	0.449	13.82 ± 0.96 (13.25–14.39)	0.762	0.458
8 wk	13.46 ± 0.65 (13.08–13.84)	0.909	13.54 ± 0.68 (13.14–13.94)	0.544	0.820
WBC count (× 10^3^/μL)					
Baseline	7.74 ± 1.81 (6.68–8.80)		7.30 ± 1.50 (6.42–8.18)		0.733
4 wk	7.43 ± 1.51 (6.54–8.32)	0.733	7.08 ± 1.25 (6.35–7.81)	0.595	0.621
8 wk	7.78 ± 1.47 (6.91–8.65)	0.733	7.18 ± 1.94 (6.04–8.32)	0.880	0.289
Neutrophil (%)					
Baseline	55.40 ± 9.70 (49.70–61.10)		53.30 ± 9.78 (47.55–59.05)		0.623
4 wk	55.30 ± 6.15 (51.69–58.91)	0.910	53.50 ± 7.95 (48.83–58.17)	0.970	0.437
8 wk	58.10 ± 8.25 (53.25–62.95)	0.404	54.10 ± 12.55 (46.72–61.48)	0.880	0.595
Lymphocyte (%)					
Baseline	39.50 ± 9.73 (33.78–45.22)		38.60 ± 2.01 (33.30–43.90)		0.762
4 wk	35.90 ± 7.72 (31.36–40.44)	0.306	38.00 ± 7.26 (33.73–42.27)	0.879	0.417
8 wk	32.30 ± 8.49 (27.31–37.29)	0.095	37.40 ± 11.74 (30.50–44.30)	0.791	0.426
Monocyte (%)					
Baseline	3.40 ± 2.91 (1.69–5.11)		3.60 ± 2.01 (2.42–4.78)		0.490
4 wk	4.10 ± 1.91 (2.98–5.22)	0.220	4.50 ± 2.42 (3.08–5.92)	0.419	0.829
8 wk	6.00 ± 2.83 (4.35–7.65)	0.128	4.80 ± 2.90 (3.10–6.50)	0.319	0.518
Eosinophil (%)					
Baseline	2.70 ± 1.70 (1.70–3.70)		3.75 ± 2.12 (2.52–4.98)		0.848
4 wk	2.80 ± 2.15 (1.54–4.06)	0.969	3.13 ± 2.59 (1.62–4.63)	0.566	0.589
8 wk	3.50 ± 1.77 (2.47–4.53)	0.848	2.56 ± 2.13 (1.31–3.80)	0.515	0.514
Basophil (%)					
Baseline	0.90 ± 1.10 (0.25–1.55)		1.38 ± 0.52 (1.07–1.68)		0.473
4 wk	1.50 ± 0.55 (1.19–1.81)	0.872	1.75 ± 1.16 (1.07–2.43)	0.807	0.434
8 wk	1.63 ± 0.74 (1.19–2.06)	0.323	1.40 ± 0.55 (1.10–3.80)	0.243	0.152
Platelet count; PLT (× 10^3^/μL)					
Baseline	277.30 ± 32.09 (258.43–296.17)		240.50 ± 60.25 (205.07–275.93)		0.273
4 wk	266.20 ± 34.46 (245.94–286.46)	0.545	236.30 ± 65.79 (197.61–274.99)	0.791	0.245
8 wk	259.60 ± 31.25 (241.23–277.97)	0.290	229.60 ± 53.63 (198.07–261.13)	0.734	0.241

Values are presented as the mean ± SD (CI).

### Metabolic and safety indices

The changes in metabolic and safety parameters, including glucose, triglyceride, total cholesterol, creatinine, total protein, aspartate aminotransferase (AST), and alanine aminotransferase (ALT), in male participants are shown in Supplementary Table 1. Symptoms and adverse events were noted at each visit. The results showed no significant difference in biochemical or toxicity characteristics among male participants within or between groups. In addition, there were no significant differences in metabolic or safety parameters between female participants within the groups or between groups, as shown in Supplementary Table 2. No patients withdrew due to adverse events, and the safety assessment parameters were within the usual range.

### Immunoglobulin levels

The effects of FCM on the immunoglobulins immunoglobulin A (IgA), immunoglobulin G (IgG), and immunoglobulin M (IgM) in male and female participants are shown in Supplementary Tables 3 and 4, respectively. Within-group analysis of immunoglobulins (Igs) revealed no significant differences at baseline or after 4 or 8 weeks of treatment in either men or women. Moreover, the immunoglobulins were similar between the groups at baseline and 8 weeks.

### T, B, and natural killer (NK) cell absolute count

**Figure 3:**
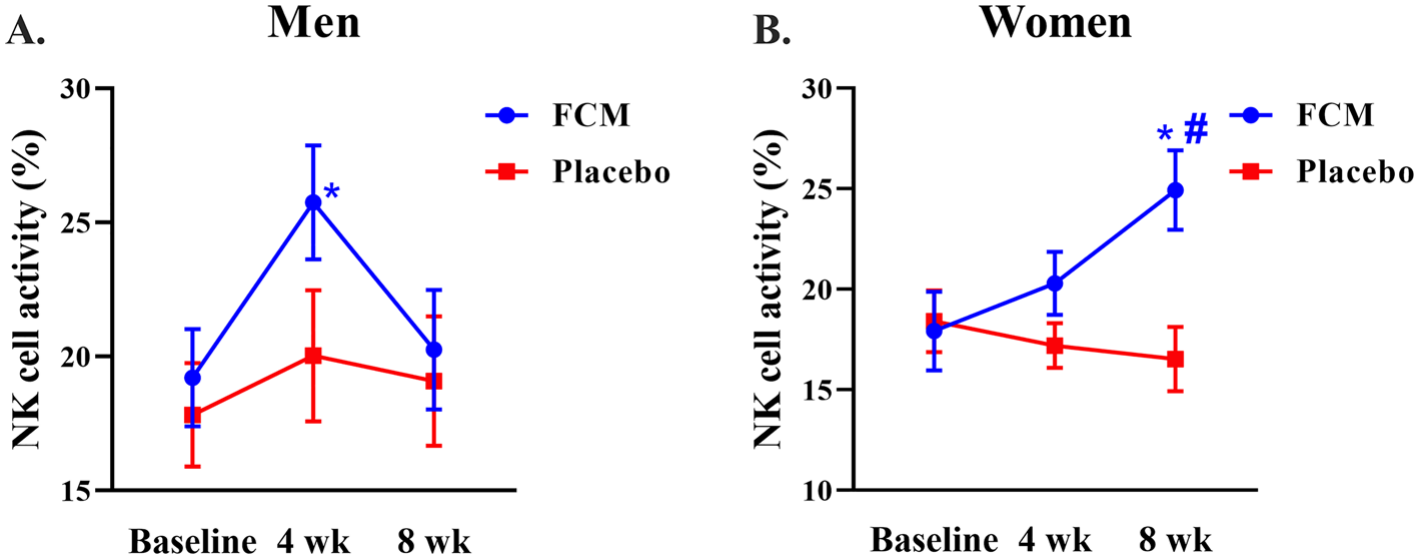
The effect of FCM on NK cell activity in men and women at weeks 0, 4, and 8. The values are presented as the means ± standard deviations (n = 10). *P < 0.05, compared with the baseline within the group. #*P* < 0.05, compared between the FCM supplement and placebo groups.

The changes in lymphocyte subsets and T, B, and NK cell counts, including those of CD3, CD4, CD8, and CD19, in male and female participants are shown in Supplementary Tables 5 and 6. There was no significant difference in these parameters between the treated and placebo groups. In addition, the numbers of CD3, CD4, CD8, and CD19 cells were similar within a group and between the groups at baseline and at the end of 8 weeks. Moreover, the effect of FCM on male and female NK cell activity was also investigated, as shown in Fig. 3. Within-group analysis indicated that NK cell activity was markedly increased in the male FCM group (*p* = 0.049) 4 weeks after receiving FCM. The rate of change in NK cell activity increased to 34.11% compared to that at baseline (Fig. 3A). Furthermore, the women who continued to consume the FCM product for 8 weeks exhibited significantly increased NK activity (*p* = 0.023) and no significant changes in other parameters. Furthermore, the changes in NK cell activity were also greater (*p* = 0.005) than those in the placebo group at a similar time point (Fig. 3B).

### Cytokine levels

**Figure 4:**
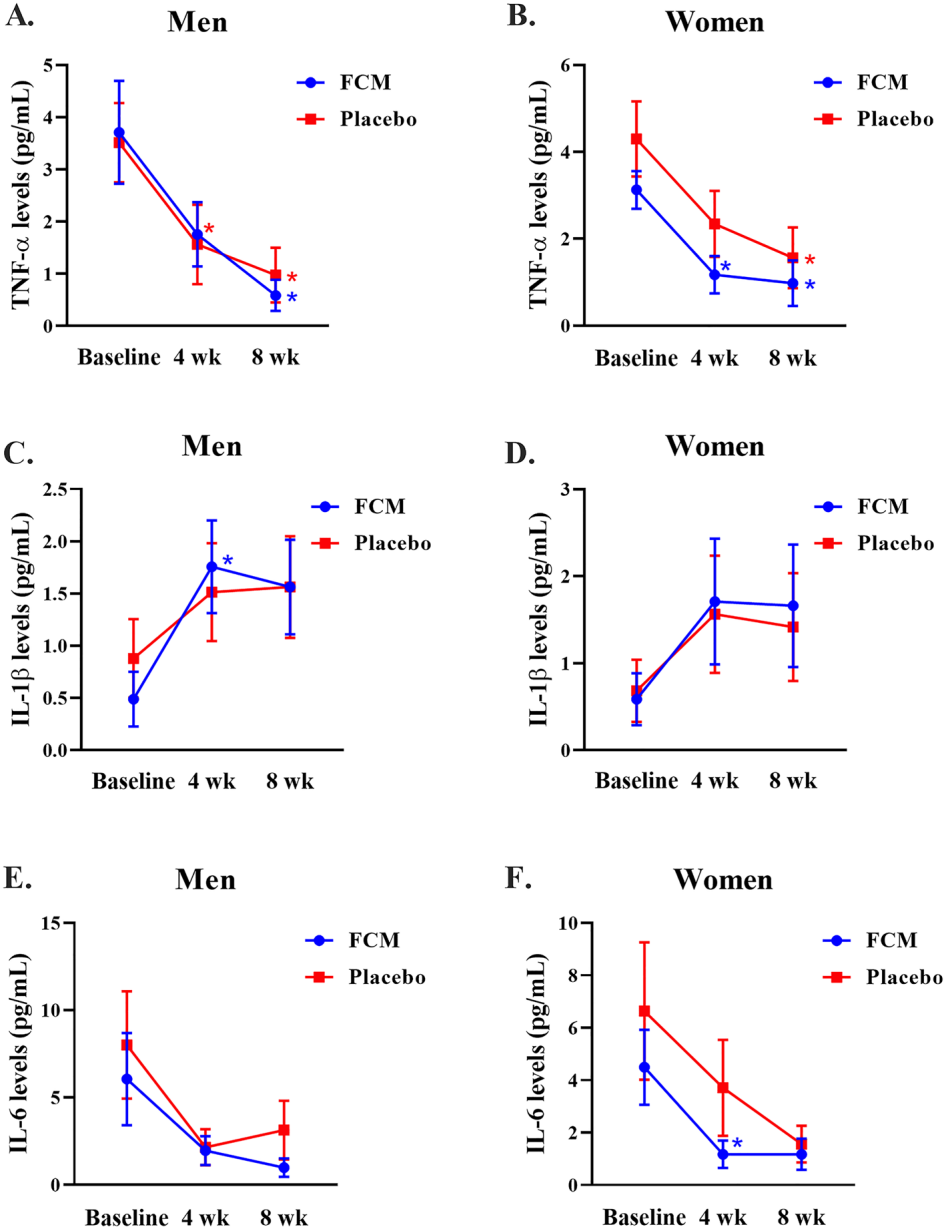
The effect of FCM on inflammatory cytokines in men and women at weeks 0, 4, and 8. The values are presented as the means ± standard deviations (n = 10). *P < 0.05, compared with the baseline within the group. #*P* < 0.05, compared between the FCM supplement and placebo groups.

The effect of FCM on inflammatory cytokine production in male and female participants is shown in Fig. 4. In male participants, the within-group analysis of the cytokines indicated that TNF-α levels (*p* = 0.047) were significantly decreased in the FCM group at the end of the 8-week study. Similarly, TNF-α levels were decreased in the placebo group at 4 (*p* = 0.018) and 8 (*p* = 0.009) weeks compared to those at baseline (Fig. 4A). In female participants, the within-group analysis revealed that TNF-α levels were lower at 4 (*p* = 0.008) and 8 (*p* = 0.008) weeks than baseline. The TNF-α levels decreased in the placebo group after receiving FCM for 8 weeks (Fig. 4B). However, within-group analysis showed that IL-1β production in the male FCM group was markedly reduced (*p* = 0.049) at the end of the 4-week study (Fig. 4C). There was no significant difference in IL-1β production among the female participants (Fig. 4D). Furthermore, IL-6 levels in male participants were not significantly different within or between groups (Fig. 4E). In addition, the within-group analysis showed that the IL-6 level decreased in the treated group (*p* = 0.047), as shown in Fig. 4F.

## Discussion

This study investigated the effect of FCM, which is rich in cordycepin, on the immune response in healthy Thai male and female participants. The results showed that NK cell activity in healthy male participants increased 4 weeks after receiving FCM compared to baseline. Furthermore, NK cell activity in healthy female participants was markedly elevated after receiving FCM for 8 weeks when compared within the group and with the placebo group. The major bioactive compounds of *C. militaris* are nucleosides such as cordycepin^16^. This compound has an immunostimulatory effect in in vitro, in vivo, and clinical studies. Cordycepin induces T-cell proliferation and immune-active lymphokine secretion in peripheral blood mononuclear cells (PBMCs)^17,18^. It enhances NK cell activity and IL-2 secretion in mouse spleen cells^19^. Additionally, cordycepin stimulated NK cell activity and the phagocytic index of monocytes in human participants^7,20^. Previous studies have reported that NK cell activity was changed after viral infection, depending on gender. There is an increase in the number of NK cells but a decrease in their function in men^21^.

This study showed that FCM activated NK cells in male and female participants at different time points. Moreover, NK cell activity was increased in FCM-treated female participants compared to that in placebo-treated participants. A previous study reported that male Korean participants had a greater absolute NK cell count and proportion than female participants due to the effect of sex hormones^22^. Furthermore, men administered estrogen plus anti-androgen agents exhibited reduced NK cell numbers^23^. Nevertheless, administering *Cordyceps sinensis* to ovariectomized rats increased estrogenic effects, such as increasing uterine weight and restoring uterine structure. Therefore, the differences in the influence of FCM on each gender might be due to sex hormones^24^. Monocytes are a component of the innate immune system and play a role in cellular homeostasis during infection and inflammatory conditions^25^. Monocytes participate in pro- and anti-inflammatory activities as part of an immune response. This study showed that monocyte levels increased in FCM-treated male participants after 4 and 8 weeks of treatment, but the increase in monocyte levels was within the normal range. These data suggested that FCM has an immunomodulatory effect.

Cytokines are signaling molecules released into the bloodstream by cells to activate or suppress an immune response. This study demonstrated that the IL-1β levels increased in the male FCM-treated group at 4 weeks compared to those at baseline. In addition, women who received FCM for 8 weeks also had elevated IL-6 levels compared to those at baseline. This result was consistent with the increase in NK cell activity in the treated group at 4 weeks (men) and 8 weeks (women) after treatment. Similarly, previous studies revealed that cordycepin treatment in macrophages increased the concentrations of IL-1β, IL-6, TNF-α, and prostaglandin-2 (PGE2) and induced nitric oxide synthase (iNOS) and cyclooxygenase-2 (COX-2) activity^5,26^. In addition, studies on healthy men indicated that supplementation with a 1.5 g/day capsule of *C. militaris* for 4 weeks increased the levels of IL-2, IL-12, NK, TNF-α, and IFN-γ, which in turn increased immunostimulatory activity^15^. These data confirmed that FCM has immune-boosting properties. However, this study was conducted with a limited number of participants. Future studies should recruit a larger number of participants.

## Conclusions

FCM stimulates the human immune response by activating NK cells, increasing monocyte concentrations, and reducing inflammatory cytokine secretion in men and women without toxicity. This beneficial effect of FCM indicates its use as a new alternative approach as a natural immunostimulatory supplement. However, it should be noted that the mechanisms underlying the immune activator effects of FCM and its impact on immune-related disease in patients require further investigation.

## Methods

### Preparation of *C. militaris* mycelium

The *C. militaris* strain used in this study was obtained from the Maejo Mushroom Learning Base, Faculty of Agricultural Production, Maejo University. The strain was cultured and maintained on potato dextrose agar, with stock cultures stored at 4 °C for preservation. For the initial inoculation in submerged fermentation, *C. militaris* mycelium was transferred to 250 ml flasks containing potato dextrose broth and then cultured at 20–25 °C for 7 days in a shaking incubator. Subsequently, the entire mycelium of *C. militaris* from each batch of scaled-up cultures was cultured in food-grade media at 20–25 °C for 7 days, homogenized and transferred to 5-L and 100-L bioreactors. The mycelia from the 100 L bioreactor were washed with distilled water and subsequently separated via centrifugation at 5000 rpm for 20 min. The mycelium was then freeze-dried at 4 °C until a constant dry weight was reached. The dried *C. militaris* mycelium was processed into powder using a grinder. The powder was stored in a zip lock bag before experimentation.

### Quantification of the active compounds of *C. militaris* powder

The cordycepin and adenosine contents of the powdered *C. militaris* mycelium were analyzed utilizing high-performance liquid chromatography—eXtra Dense Bonding (HPLC-XDB). The sample (1 g) was initially dissolved in a 15% v/v methanol solution (10 ml) and subjected to ultrasonic extraction at 60 °C for 3 h. After extraction, the mixture was centrifuged at 5000 rpm for 20 min to separate the supernatant. The supernatant was then filtered and analyzed by HPLC-XDB using a C18 column at a 1.0 ml/min flow rate. The mobile phase comprised acetonitrile and 50 mm phosphate buffer (2:98), and detection was performed at a wavelength of 254 nm using a UV‒visible detector. Standard calibration curves were constructed using various concentrations of cordycepin and adenosine (50, 100, 150, 200, and 250 μg/mL).

### Preparation of FCM for clinical assessment

FCM was prepared by blending *C. militaris* powder with fruit juice at 90 °C for 10 min, after which the mixture was immediately bottled and sealed. Subsequently, the beverage underwent sterilization through a retort at 121 °C for 20 min. The concentration of cordycepin in 75 ml of FCM was not less than 2.85 mg/portion according to a previous study^15^, and no bacteria, such as *Escherichia coli*, *Salmonella spp.*, or *Staphylococcus aureus*, were detected*.* The placebo beverage consisted of 75 mL of date palm fruit juice without the addition of *C. militaris*.

### Sample size calculation

The sample size in this study was calculated based on previously described changes in C-reactive protein (CRP), which is an immune stimulation marker^27^. The formula was n_1_ = (Z_a_ + Z_b_)^2^ × s^2^ x (r + 1)/(μ_0_—μ_1_)^2^ × r, where r = n_0_/n_1_. The change in CRP from 640.87 ± 263.21 to 475.06 mg/dL was attributed to the use of antidepressant drugs^28^. Thus, the sample size was 39.512.

### Ethical consideration

This study followed the National Ethics Committee Accreditation System of Thailand (NECAST) guidelines. The University of Phayao Human Ethics Committee (UP-HEC 1.3/026/65, Phayao, Thailand) approved the human study, and the clinical trial was registered with the WHO International Clinical Trial Registry Platform (03/10/2023, registration number: TCTR20231003001; https://www.thaiclinicaltrials.org/show/TCTR20231003001) and ClinicalTrials.gov (18/11/2023, registration number: NCT06138444; https://clinicaltrials.gov/study/NCT06138444).

### Study participants

Healthy Thai men and women aged 25–60 years were recruited at the School of Medical Sciences, University of Phayao, in 2022. Written informed consent was obtained from all research participants. A total of 40 participants were randomly assigned to one of the study groups (10 participants each). The inclusion criteria were as follows: (1) male and female adult participants aged 25–60 years during the screening test; (2) no history of hypersensitivity or idiosyncratic reactions to drugs or herbal products; and (3) willingness to participate in the project throughout the research program. The exclusion criteria were as follows: (1) participants who were diagnosed with immune-mediated disease, nervous system disorders, cardiovascular disease, or liver or kidney disease; (2) participants who were diagnosed with chronic health problems such as hypertension, diabetes, or renal failure; (3) a body mass index (BMI) > 29.9 or < 18 kg/m^2^; (4) participants who were pregnant, lactating or intended to become pregnant during the trial period; (5) participants who, within two weeks, ingested a drug or functional food that may affect the immunomodulatory effect of the test product; and (6) participants who had an alanine transaminase (ALT) or aspartate transaminase (AST) plasma level more than three times the guideline of the organization. Laboratory research, including hematology, serum biochemistry, blood coagulation, and urinalysis, was conducted to confirm the eligibility of the research participants. The participants who met the inclusion requirements were randomized into experimental groups. Twenty random numbers were generated using SPSS 26.0. The 1st–10th digits of each gender were assigned to the FCM group, and the remaining 11th–20th digits of each gender were assigned to the placebo group. All researchers, participants, and related medical staff were blinded to the intervention assignments throughout the study.

### Intervention

This study was separated into 3 visits (0–3) over 8 weeks of the trial, as shown in Fig. 1. After screening 62 participants using the medical history questionnaire, all volunteers received screening procedures at visit 0 (screening/baseline). The laboratory reports revealed that 40 participants (20 male and 20 female participants) met all the eligibility criteria. The participants were randomized into 4 groups at visit 1. Group 1: Ten male participants were randomized to receive a single oral dose of FCM containing 2.85 mg of cordycepin per portion. Group 2: Ten male participants were randomized to the placebo group. Group 3: Ten female participants were randomized to receive a single oral dose of FCM. Group 4: Ten female participants were randomized to the placebo group. The participants received either 75 mL of FCM or a placebo (once daily) after breakfast. During the 60 days of the intervention, the participants were asked to keep their usual diet and were prohibited from consuming any functional foods or dietary supplements. Physical examination, vital signs, and adverse reactions were investigated at each visit.

### Biochemical analyses

At the screening visit (visit 0), each participant was given a screening number, and the participants' demographic information, medical history, and medication information were examined. The first visit was conducted within one week after screening. Test substances were packaged for 2 weeks according to a randomized protocol and were prescribed to participants in the order of the first visit. Every 15 days after taking the test substance, the participants were asked to report any adverse events newly prescribed after returning the remaining test substance. Blood samples (10 ml for each time point) were collected at 0, 30, and 60 days. The fasting blood samples were collected into plain and EDTA tubes and delivered to the laboratory within 2 h of collection. This experiment examined the changes in immune cell markers (natural killer cells (NK cells), cluster of differentiation 3 (CD3), cluster of differentiation 4 (CD4), cluster of differentiation 8 (CD8), B-lymphocyte antigen CD19 (CD19)), and immunoglobulins (immunoglobulin A (IgA), immunoglobulin G (IgG), and immunoglobulin M (IgM)). Moreover, the safety parameters were determined by measuring the complete blood count (CBC) and fasting blood glucose, triglyceride, total cholesterol, creatinine, total protein, aspartate aminotransferase (AST), and alanine aminotransferase (ALT) levels at the University of Phayao Hospital, School of Medicine, University of Phayao and National Healthcare System Co., Ltd., Bangkok Hospital Chiang Rai.

### Inflammatory cytokine assay

ELISA was used to determine the impact of FCM on the levels of proinflammatory cytokines. Blood samples were collected from the experimental groups at each visit. TNF-α, IL-1β, and IL-6 levels were measured using ELISA kits (BioLegend, CA, USA). Cytokine levels were calculated and are expressed as pg/mL.

### Statistical analysis

The data are expressed as the mean ± standard deviation (SD) (confidence interval CI)). Statistical differences were assessed using the nonparametric Mann‒Whitney U test for nonnormally distributed variables within and between groups. Statistical analyses were conducted using the Statistical Package for IBM SPSS Statistical Software version 26 (IBM Corp., NY, USA). Differences were considered significant when *p* < 0.05.

### Supplementary Information

Supplementary Tables.

## Data Availability

The datasets generated and/or analyzed during the current study are not publicly available to protect participants confidentiality but are available from the corresponding author upon reasonable request.
