# Supplementary material for: A randomized controlled clinical trial examining the effects of Cordyceps militaris beverage on the immune response in healthy adults
Source: Sci Rep. 2024 Apr 5;14:7994. doi: 10.1038/s41598-024-58742-z (PMC10997757; doi:10.1038/s41598-024-58742-z)
Supplement: Supplementary file 1 — Supplementary Tables. [file 41598_2024_58742_MOESM1_ESM.docx]

**Supplementary Table 1** Comparison of the metabolic and safety parameters level in serum samples of men supplemented with FCM and placebo group at weeks 0, 4, and 8.

| **Outcome variable** | **FCM supplemented group (n=10) mean ±SD (CI)** | | | | | **Placebo group (n=10) mean ±SD (CI)** | | ***P* value^#^** |
| --- | --- | --- | --- | --- | --- | --- | --- | --- |
| **Glucose (mg/dL)** |  | | | | *p value** |  | *p value** |  |
| Baseline | 96.10 ±12.03 (89.03-103.17) | | | |  | 100.70±30.73 (82.63-118.77) |  | 0.791 |
| 4 wk | 91.60 ±8.09 (86.85-96.35) | | | | 0.596 | 102.50±28.04 (86.01-128.14) | 0.364 | 0.198 |
| 8 wk | 93.00±6.07 (89.43-96.57) | | | | 0.909 | 108.50±38.50 (82.86-128.14) | 0.623 | 0.820 |
| **Triglyceride (mg/dL)** |  | | | |  |  |  |  |
| Baseline | 114.80±42.80 (89.63-139.97) | | | |  | 130.20±37.00 (108.45-151.95) |  | 0.257 |
| 4 wk | 116.70±53.13 (85.46-147.94) | | | | 0.850 | 113.70±51.12 (83.64-143.76) | 0.473 | 0.880 |
| 8 wk | 100.80±41.18 (76.59-125.01) | | | | 0.520 | 136.50±48.89 (107.75-165.25) | 1.000 | 0.070 |
| **Total Cholesterol (mg/dL)** | |  | | |  |  |  |  |
| Baseline | 182.60±35.95 (161.46-203.74) | | | |  | 174.30±37.98 (151.97-196.63) |  | 0.677 |
| 4 wk | 181.50±30.33 (163.67-199.33) | | | | 0.970 | 184.60±41.87 (159.98-209.22) | 0.520 | 0.850 |
| 8 wk | 172.40±31.42 (153.93-190.87) | | | | 0.427 | 161.60±21.45 (148.99-174.21) | 0.345 | 0.734 |
| **Creatinine (mg/dL)** |  | | | |  |  |  |  |
| Baseline | 1.10±0.16 (1.01-1.20) | | | |  | 1.06±0.08 (1.01-1.10) |  | 0.520 |
| 4 wk | 1.05±0.16 (0.96-1.15) | | | | 0.520 | 0.99± (0.93-1.05) | 0.130 | 0.445 |
| 8 wk | 1.09±0.17 (0.99-1.19) | | | | 0.970 | 0.98±0.10 (0.92-1.04) | 0.121 | 0.088 |
| **Total Protein; TP (g/dL)** | | |  | |  |  |  |  |
| Baseline | 7.37±0.33 (7.18-7.56) | | | |  | 7.37±0.57 (7.04-7.70) |  | 0.939 |
| 4 wk | 7.46±0.49 (7.17-7.75) | | | | 0.621 | 7.51±0.43 (7.26-7.76) | 0.518 | 0.790 |
| 8 wk | 7.63±0.37 (7.38-7.88) | | | | 0.148 | 7.56±0.37 (7.34-7.78) | 0.404 | 0.704 |
| **Aspartate aminotransferase; AST (U/L)** | | | |  |  |  |  |  |
| Baseline | 31.40±19.27 (20.07-42.73) | | | |  | 32.10±6.64 (28.20-36.00) |  | 0.240 |
| 4 wk | 28.10±11.88 (21.11-35.09) | | | | 0.849 | 29.70±7.17 (25.49-33.91) | 0.184 | 0.381 |
| 8 wk | 30.40±16.40 (20.76-40.04) | | | | 0.850 | 29.30±9.52 (23.40-35.20) | 0.095 | 0.819 |
| **Alanine aminotransferase; ALT (U/L)** | | | |  |  |  |  |  |
| Baseline | 29.80±12.82 (22.26-37.34) | | | |  | 36.10±11.54 (29.31-42.89) |  | 0.139 |
| 4 wk | 33.50±20.93 (21.19-45.81) | | | | 0.940 | 37.70±14.98 (28.89-46.51) | 0.879 | 0.343 |
| 8 wk | 28.60±14.18 (20.27-36.93) | | | | 0.940 | 33.90±9.19 (28.49-39.31) | 0.495 | 0.307 |

Values are presented as the mean±SD (CI)

**Supplementary Table 2** Comparison of the level of metabolic and safety parameters in serum samples of women supplemented with FCM and placebo group at weeks 0, 4, and 8.

| **Outcome variable** | **FCM supplemented group (n=10) mean ±SD (CI)** | | | | | | **Placebo group (n=10) mean ±SD (CI)** | | ***p value^#^*** |
| --- | --- | --- | --- | --- | --- | --- | --- | --- | --- |
| **Glucose (mg/dL)** |  | | | | | *p value** |  | *p value** |  |
| Baseline | 91.10±8.24 (86.26-95.94) | | | | |  | 92.40±6.06 (88.84-95.96) |  | 0.470 |
| 4 wk | 89.20±15.21 (80.26-98.14) | | | | | 0.224 | 91.50±6.10(87.92-95.08) | 0.704 | 0.161 |
| 8 wk | 93.50±12.09 (86.39-100.61) | | | | | 0.676 | 93.20±4.26 (90.69-95.71) | 0.733 | 0.940 |
| **Triglyceride (mg/dL)** | |  | | | |  |  |  |  |
| Baseline | 101.30±40.56 (77.45-125.15) | | | | |  | 93.20±41.60 (68.74-117.66) |  | 0.545 |
| 4 wk | 118.80±52.47 (87.95-149.65) | | | | | 0.496 | 96.20±35.47 (75.35-117.05) | 0.762 | 0.427 |
| 8 wk | 95.56±34.01 (75.68-115.43) | | | | | 0.880 | 90.60±31.48 (72.09-109.11) | 0.970 | 0.821 |
| **Total Cholesterol (mg/dL)** | | |  | | |  |  |  |  |
| Baseline | 187.30±22.69 (173.96-200.64) | | | | |  | 176.40±55.43 (143.81-208.99) |  | 0.880 |
| 4 wk | 176.80±30.46 (158.89-194.71) | | | | | 0.199 | 162.50±45.35 (135.83-189.17) | 0.545 | 0.344 |
| 8 wk | 167.70±21.17 (155.25-180.15) | | | | | 0.112 | 160.40±40.51 (136.58-184.22) | 0.450 | 0.677 |
| **Creatinine (mg/dL)** | | |  | | |  |  |  |  |
| Baseline | 0.76±0.11 (0.69-0.82) | | | | |  | 0.79±0.11 (0.73-0.86) |  | 0.344 |
| 4 wk | 0.68±0.08 (0.63-0.73) | | | | | 0.169 | 0.72±0.09 (0.67-0.78) | 0.197 | 0.273 |
| 8 wk | 0.67±0.15 (0.58-0.76) | | | | | 0.111 | 0.72±0.13 (0.64-0.79) | 0.185 | 0.405 |
| **Total Protein; TP (g/dL)** | | |  | | |  |  |  |  |
| Baseline | 7.56±0.33 (7.36-7.76) | | | | |  | 7.31±0.35 (7.11-7.51) |  | 0.195 |
| 4 wk | 7.42±0.38 (7.20-7.64) | | | | | 0.305 | 7.28±0.27 (7.12-7.44) | 0.673 | 0.465 |
| 8 wk | 7.74±0.36 (7.53-7.95) | | | | | 0.237 | 7.62±0.38 (7.40-7.84) | 0.074 | 0.675 |
| **Aspartate aminotransferase; AST (U/L)** | | | | |  |  |  |  |  |
| Baseline | 25.30±5.33 (22.16-28.44) | | | | |  | 23.30±6.50 (19.48-27.12) |  | 0.705 |
| 4 wk | 22.20±5.41 (19.02-25.38) | | | | | 0.289 | 22.80±4.10 (20.39-25.21) | 0.567 | 0.970 |
| 8 wk | 23.00±12.03 (15.93-30.07) | | | | | 0.069 | 23.60±7.43 (19.23-27.97) | 0.733 | 0.426 |
| **Alanine aminotransferase; ALT (U/L)** | | | |  | |  |  |  |  |
| Baseline | 22.80±15.35 (13.77-31.83) | | | | |  | 25.30±20.51 (13.24-37.36) |  | 0.879 |
| 4 wk | 20.20±10.27 (14.16-26.24) | | | | | 0.704 | 24.10±20.45 (12.07-36.13) | 0.518 | 0.732 |
| 8 wk | 23.60±16.89 (13.67-33.53) | | | | | 0.733 | 25.20±12.16 (18.05-32.35) | 0.732 | 0.496 |

Values are presented as the mean±SD (CI)

**Supplementary Table 3** Comparison of the level of immunoglobulins in serum samples of men supplemented with FCM and placebo group at weeks 0, 4, and 8.

| **Outcome variable** | **FCM supplemented group (n=10) mean ±SD (CI)** | | **Placebo group (n=10) mean ±SD (CI)** | | ***p value^#^*** |
| --- | --- | --- | --- | --- | --- |
| **IgA (mg/dL)** |  | *p value** |  | *p value** |  |
| Baseline | 252.50±63.33 (215.26-289.74) |  | 261.20±86.68 (210.23-312.17) |  | 0.940 |
| 4 wk | 267.80±71.79 (225.59-310.01) | 0.597 | 271.20±93.08 (216.47-325.93) | 0.705 | 0.880 |
| 8 wk | 259.60±69.72 (218.61-300.59) | 0.821 | 263.20±87.70 (211.63-314.77) | 1.000 | 0.910 |
| **IgG (mg/dL)** |  |  |  |  |  |
| Baseline | 1423.00±177.77 (1318.47-1527.53) |  | 1420.00±254.12 (1270.58-1569.42) |  | 0.705 |
| 4 wk | 1441.00±204.15 (1320.96-1561.04) | 0.970 | 1457.00±256.78 (1306.02-1607.98) | 0.762 | 0.910 |
| 8 wk | 1383.00±178.39 (1278.11-1487.89) | 0.545 | 1398.00-259.91 (1245.18-1550.82) | 0.910 | 0.910 |
| **IgM (mg/dL)** |  |  |  |  |  |
| Baseline | 76.22±20.31 (64.57-87.87) |  | 97.81±23.93 (83.82-111.80) |  | 0.140 |
| 4 wk | 79.01±22.42 (66.14-91.88) | 0.791 | 99.69±23.03 (86.23-113.14) | 0.545 | 0.212 |
| 8 wk | 71.22±22.92 (58.07-84.37) | 0.406 | 90.10±26.50 (74.62-105.58) | 0.199 | 0.290 |

Values are presented as the mean±SD (CI)

**Supplementary Table 4** Comparison of the level of immunoglobulins in serum samples of women supplemented with FCM and placebo group at weeks 0, 4, and 8.

| **Outcome variable** | **FCM supplemented group (n=10) mean ±SD (CI)** | | **Placebo group (n=10) mean ±SD (CI)** | | ***p value^#^*** |
| --- | --- | --- | --- | --- | --- |
| **IgA (mg/dL)** |  | *p value** |  | *p value** |  |
| Baseline | 239.40±80.77 (191.91-286.89) |  | 256.60±60.79 (220.85-292.35) |  | 0.762 |
| 4 wk | 230.50±78.14 (184.55-276.45) | 0.734 | 266.10±66.83 (226.80-305.40) | 0.677 | 0.326 |
| 8 wk | 230.50±73.02 (187.56-273.44) | 0.821 | 263.30±63.75 (225.82-300.78) | 0.821 | 0.326 |
| **IgG (mg/dL)** |  |  |  |  |  |
| Baseline | 1481.00±125.47 (1407.22-1554.78) |  | 1450.00±149.96 (1361.82-1538.18) |  | 0.596 |
| 4 wk | 1457.00±125.70 (1383.09-1530.91) | 0.623 | 1439.00±162.72 (1343.32-1534.68) | 0.880 | 0.880 |
| 8 wk | 1449.00±150.37 (1360.58-1537.42) | 0.496 | 1442.00±138.15 (1360.77-1523.23) | 0.970 | 1.000 |
| **IgM (mg/dL)** |  |  |  |  |  |
| Baseline | 125.18±61.09 (87.16-155.92) |  | 119.78±56.38 (86.63-152.93) |  | 0.850 |
| 4 wk | 121.54±58.47 (87.16-155.92) | 0.970 | 119.89±56.57 (86.63-153.15) | 1.000 | 0.940 |
| 8 wk | 116.85±55.75 (84.07-149.63) | 0.734 | 112.39±54.98 (80.06-144.72) | 0.545 | 0.734 |

Values are presented as the mean±SD (CI)

**Supplementary Table 5** Comparison of TBNK absolute count in serum samples of men supplemented with FCM and placebo group at weeks 0, 4, and 8.

| **Outcome variable** | **FCM supplemented group (n=10) mean ±SD (CI)** | | **Placebo group (n=10) mean ±SD (CI)** | | ***p value^#^*** |
| --- | --- | --- | --- | --- | --- |
| **CD3 (%)** |  | *p value** |  | *p value** |  |
| Baseline | 63.79±6.94 (59.71-67.87) |  | 66.17±7.51 (61.75-70.59) |  | 0.406 |
| 4 wk | 60.89±6.72 (56.94-64.85) | 0.545 | 63.45±10.63 (57.20-69.70) | 0.496 | 0.597 |
| 8 wk | 61.67±7.38 (57.33-66.01) | 0.450 | 63.47±10.08 (57.55-69.40) | 0.496 | 0.705 |
| **CD4 (%)** |  |  |  |  |  |
| Baseline | 28.66±6.43 (24.87-32.44) |  | 32.07±5.55 (28.80-35.33) |  | 0.326 |
| 4 wk | 27.31±6.16 (23.68-30.93) | 0.650 | 30.06±5.57 (26.79-33.33) | 0.406 | 0.226 |
| 8 wk | 28.92±6.48 (25.11-32.73) | 1.000 | 30.88±5.77 (27.49-34.28) | 0.650 | 0.364 |
| **CD8 (%)** |  |  |  |  |  |
| Baseline | 28.19±7.83 (23.58-32.79) |  | 28.43±5.03 (25.47-31.38) |  | 0.326 |
| 4 wk | 27.15±7.30 (22.86-31.44) | 0.650 | 28.00±7.04 (23.86-32.14) | 0.821 | 0.226 |
| 8 wk | 27.13±7.64 (22.63-31.62) | 0.496 | 27.93±5.68 (24.59-31.27) | 0.880 | 0.364 |
| **CD4:CD8** |  |  |  |  |  |
| Baseline | 1.10±0.45 (0.84-1.37) |  | 1.17±0.35 (0.96-1.37) |  | 0.472 |
| 4 wk | 1.08±0.42 (0.83-1.33) | 0.910 | 1.14±0.39 (0.91-1.37) | 0.545 | 0.496 |
| 8 wk | 1.17±0.49 (0.88-1.45) | 0.762 | 1.14±0.26 (0.99-1.29) | 0.791 | 0.623 |
| **CD19 (%)** |  |  |  |  |  |
| Baseline | 12.64±4.44 (10.03-15.25) |  | 13.31±2.15 (12.05-14.58) |  | 0.364 |
| 4 wk | 12.10±3.16 (10.24-13.96) | 0.821 | 13.86±4.32 (11.32-16.40) | 0.910 | 0.226 |
| 8 wk | 12.56±4.08 (10.15-14.96) | 0.880 | 12.67±2.33 (11.30-14.04) | 0.326 | 0.650 |

Values are presented as the mean±SD (CI)

**Supplementary Table 6** Comparison of T and B Natural Killer Cells in serum samples of women supplemented with FCM and placebo group at weeks 0, 4, and 8.

| **Outcome variable** | **FCM supplemented group (n=10) mean ±SD (CI)** | | **Placebo group (n=10) mean ±SD (CI)** | | ***p value^#^*** |
| --- | --- | --- | --- | --- | --- |
| **CD3 (%)** |  | *p value** |  | *p value** |  |
| Baseline | 63.64±5.89 (60.17-67.10) |  | 67.68±5.67 (64.35-71.01) |  | 0.174 |
| 4 wk | 64.77±5.31 (61.65-67.89) | 0.650 | 67.80±5.05 (64.83-70.78) | 0.705 | 0.273 |
| 8 wk | 60.76±15.01 (51.93-69.59) | 0.762 | 67.72±6.83 (63.70-71.73) | 0.650 | 0.112 |
| **CD4 (%)** |  |  |  |  |  |
| Baseline | 37.47±6.75 (33.50-41.43) |  | 36.57±8.34 (31.67-41.47) |  | 0.705 |
| 4 wk | 39.38±4.36 (36.81-41.94) | 0.597 | 34.83±6.17 (31.20-38.46) | 0.762 | 0.257 |
| 8 wk | 39.02±7.23 (34.77-43.27) | 0.545 | 33.94±8.88 (28.72-39.16) | 0.364 | 0.650 |
| **CD8 (%)** |  |  |  |  |  |
| Baseline | 21.76±4.09 (19.35-24.17) |  | 27.86±6.84 (23.84-31.88) |  | 0.705 |
| 4 wk | 21.60±4.15 (19.16-24.04) | 0.910 | 27.28±7.31 (22.98-31.58) | 0.880 | 0.257 |
| 8 wk | 20.74±4.15 (18.30-23.18) | 0.450 | 25.41±6.01 (21.87-28.94) | 0.326 | 0.650 |
| **CD4:CD8** |  |  |  |  |  |
| Baseline | 1.81±0.57 (1.47-2.14) |  | 1.41±0.59 (1.07-1.76) |  | 0.082 |
| 4 wk | 1.90±0.48 (1.62-2.18) | 0.705 | 1.49±0.64 (1.12-1.87) | 0.734 | 0.096 |
| 8 wk | 1.98±0.63 (1.61-2.35) | 0.545 | 1.54±0.59 (1.19-1.89) | 0.257 | 0.070 |
| **CD19 (%)** |  |  |  |  |  |
| Baseline | 12.60±3.46 (10.57-14.63) |  | 12.59±3.51 (10.53-14.66) |  | 0.880 |
| 4 wk | 13.21±2.09 (11.98-14.43) | 0.450 | 12.60±4.26 (10.09-15.10) | 0.821 | 0.496 |
| 8 wk | 14.01±3.45 (11.98-16.03) | 0.496 | 13.15±3.48 (11.11-15.20) | 0.650 | 0.496 |

Values are presented as the mean±SD (CI)
